# Nasopharyngeal aspirates in children with severe community-acquired pneumonia collected within 3 days before bronchoscopy can partially reflect the pathogens in bronchoalveolar lavage fluids

**DOI:** 10.1186/s12879-022-07749-w

**Published:** 2022-11-01

**Authors:** Qiguo Zhu, Junli Zhou, Fei Li, Peng Shi, Yi Lu, Xiaoliang Lin, Lin Yuan, Zhiqiang Zhuo, Jun Shen

**Affiliations:** 1 Xiamen Children’s Hospital, Xiamen Branch of children’s Hospital of Fudan University, 361006 Xia Men, China; 2 Infectious Disease Department, Children’s Hospital of Fudan University, National Children’s Medical Center, 201102 Shanghai, China; 3 Statistics and data management center, Children’s Hospital of Fudan University, National Children’s Medical Center, 201102 Shanghai, China

**Keywords:** Bronchoalveolar lavage fluid, Nasopharyngeal aspirate, Severe community-acquired pneumonia, Etiology, Children

## Abstract

**Background:**

There is little evidence about consistency between nasopharyngeal and pulmonary pathogens in children with severe pneumonia. This study aims to compare the difference of pathogens between nasopharyngeal aspirates (NPAs) collected before bronchoscopy and bronchoalveolar lavage fluids (BALFs) in children with severe community-acquired pneumonia (SCAP).

**Methods:**

NPAs and BALFs were collected form pediatric SCAP cases hospitalized from January 2018 to March 2019. NPAs were colleced within 3 days before bronchoscopy. Samples were detected by direct immunofluorescence assay (DFA) for seven respiratory viruses and by routine bacterial culture in the clinical microbiology laboratory. Respiratory syncytial virus (RSV), Adenovirus (ADV), Influenza virus types A, B (IV-A and IV-B), Parainfluenza virus 1–3 (PIV1-3) were detected with a commercial assay. The virological and bacteriological detention results of NPAs were compared with the results of BALFs.

**Results:**

In total 204 cases with mean age of 3.4 ± 2.8 years (IQR, 1 month-14 years) were included in the study. Both NPA and BALF were collected from those cases. The positive rates of pathogen in NPAs and BALFs were 25.0% (51/204) and 36.7% (75/204), respectively (x^2^ = 6.614, P = 0.010). Respiratory viruses were found in 16.1% (33/204) from NPAs and 32.3% (66/204) from BALFs (x^2^ = 14.524, P < 0.001). RSV and ADV were the two most frequent detected viruses in NPAs and BALFs. High consistentcy of pathogens between NPAs and BALFs was observed, and 96.9% (32/33) viruses detected in NPAs were also found in BALFs. While bacteria were isolated from 12.7% (26/204) and 10.7% (22/204) of the two kinds of samples, respectively (x^2^ = 0.378, *P* = 0.539). In addition, Haemophilus influenzae (HI) was the dominant germ in both samples.

**Conclusion:**

The DFA method used to detect seven respiratory viruses from NPAs collected within 3 days before bronchoscopy can partially reflect the pathogens in the lungs in children with SCAP.

**Supplementary Information:**

The online version contains supplementary material available at 10.1186/s12879-022-07749-w.

## Background

SCAP is the main cause of death of children under 5 years old [1, 2]. Rapid identification of pathogens and targeted treatments could help to save lives. BALFs collected by flexible bronchoscope are ideal samples for identifing pulmonary etiology, but few cases expericed such an invasive procedure [3–5]. Prior to obtaining the etiological results from BALFs, clinical empiric anti-infection treatments usually rely on the findings from other samples, including NPAs collected from upper respiratory tracts [6–8]. Whether the detection results of upper respiratory tract samples could not reflect the pathogens in the lungs, caused the clinicians fail to prescribe appropriate antibiotics and led to the diseases progress was unknown. The comparison of pathogens detected between NPAs and BALs has not been adequately explored or studied [9, 10]. In this study, we retrospectively analyzed the etiology reports of NPAs before bronchoscopy and BALFs from the same cases with SACP admitted to our hospital from January 2018 to March 2019.

## Methods

### Study population

Patients < 18 years hospitalized in Xiamen Children’s Hospital from January 2018 to March 2019 due to SCAP and experiened bronchoscopy operation were retrospectively studied. Inclusion criteria: [1] Met the diagnostic criteria of SCAP and BALFs; SCAP met the definition of 2011 Infectious Diseases Society of America community-acquired pneumonia management guide line. That is, CAP cases need invasive mechanical ventilation, with multi-lobar infiltrates or presence of effusion, etc. [11]; [2] NPAs were collected within 3 days before bronchoscopy operation; [3] Both NPAs and BALFs from the same case were send to the clinical microbiology Laboratory for DFA and routine bacterial culture. Exclusion criteria: [1] Only NPAs or BALFs were collected; [2] NPAs were collected more than 3 days before bronchoscopy operation; [3] Patients with hospital acquired pneumonia (HAP) and aspiration pneumonia. HAP was defined as pneumonia that occurs 48 h or more after hospital admission and not incubating at the admission time.

The criteria or indication of bronchoscopy in our medical center is based on the “Guideline for diagnosis and treatment of community-acquired pneumonia in Children (2019 version), Chinese Journal of Clinical Infectious Diseases, 2019,12[1]:6–13”: Patient has persistent clinical symptoms, lung imaging suggests significant pulmonary consolidation or atelectasis; empiric anti-infective therapy fails to show significant improvement. The purpose of performing bronchoscopy is to remove the mass of secretions blocking the airway and to examine possible airway foreign bodies or congenital malformations. So, the invasive procedure of bronchoscopy is used both for diagnostic and therapeutic purposes in most of cases [5, 11].

### Pathogens detection

**Fig. 1:**
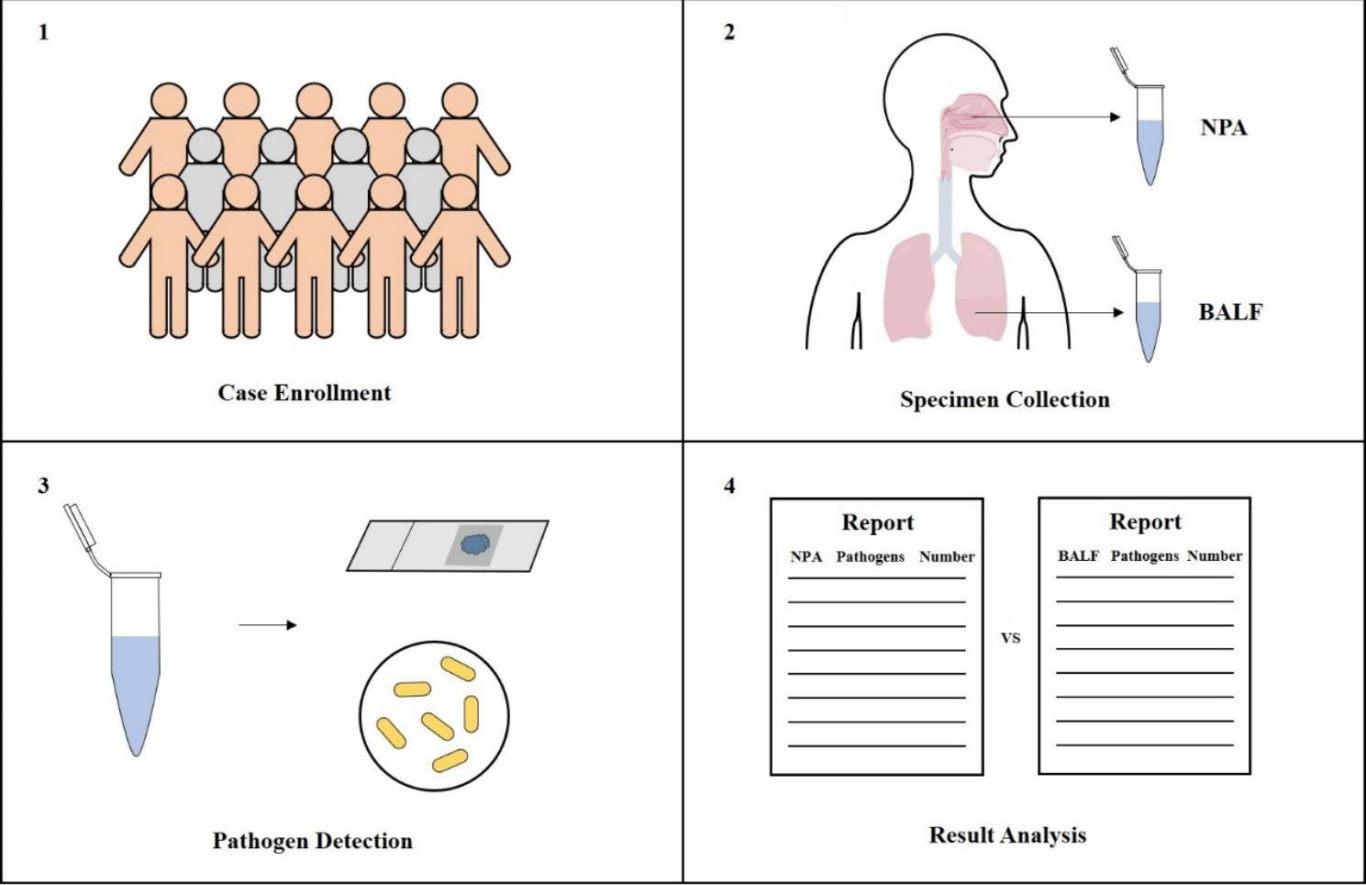
Flowchart to enroll the cases and collect samples sent for pathogens detection in this study. Both NPAs and BALFs from the same case were tested by DFA and routine bacterial culture

RSV, ADV, IV-A, IV-B and PIV1-3 were detected by a commercial assay (Chemicon International Inc, Temecula, CA, USA) as previous reported [12]. Samples were sent to bench as soon as posobile once collected for routine bacterial culture (Fig. 1). The NPAs and BALFs were inoculated onto tryptone soya blood agar supplemented with 5 µg gentamicin and incubated at 37 °C under 5% CO2 atmosphere overnight (16–20 h). The plate with no growth after 24 h of incubation was further incubated for additional 16–20 h. Isolates were identified which included hemolytic property, Gram’s staining, catalase, optochin sensitivity, and bile solubility tests.

### Clinical data collection

Clinical data was obtained from hospital information system. Patients’ information included age, gender, dates of the samples collected and bronchoscopy operation, and the diagnosis when discharged.

### Statistical analysis

Continuous variables were presented as mean ± standard deviation (SD), and compared by Student t test or median (IQR, inter-quartile range), and non-parametric tests according to the distribution of the data. Categorical variables were expressed as number (%) and analyzed by chi-squared tests or Fisher’s exact tests, when appropriate. Kappa test was used to compare the consistency of pathogens detection between BALFs and NPAs. Taking the BALFs pathogens detection results as the “golden standard”, the positive predictive value (PPV) and negative predictive value (NPV) of NPAs pathogens were calculated. The SPSS 24.0 was used to analyze the data and the significance level was set at P < 0.05.

## Results

### Demographic characteristics

In total, 240 cases were enrolled in the study. Thirty-six cases were excluded due to either NPAs collected 3 days before bronchoscopy or on the day of BALFs collected. Flnally 204 cases were included in the analysis, with mean age of 3.4 ± 2.8 years ( IQR, 1 month-14 years ). In terms of age distribution, 24.0% (49/204), 27.9% (57/204), 27.4% (56/204) and 20.6% (42/204) of the cases were under one year old, ≥ 1 year old and < 3 years old, ≥ 3 years old and < 6 years old, and ≥ 6 years old, respectively (Supplement Table).

### Pathogens detected from NPAs

**Fig. 2:**
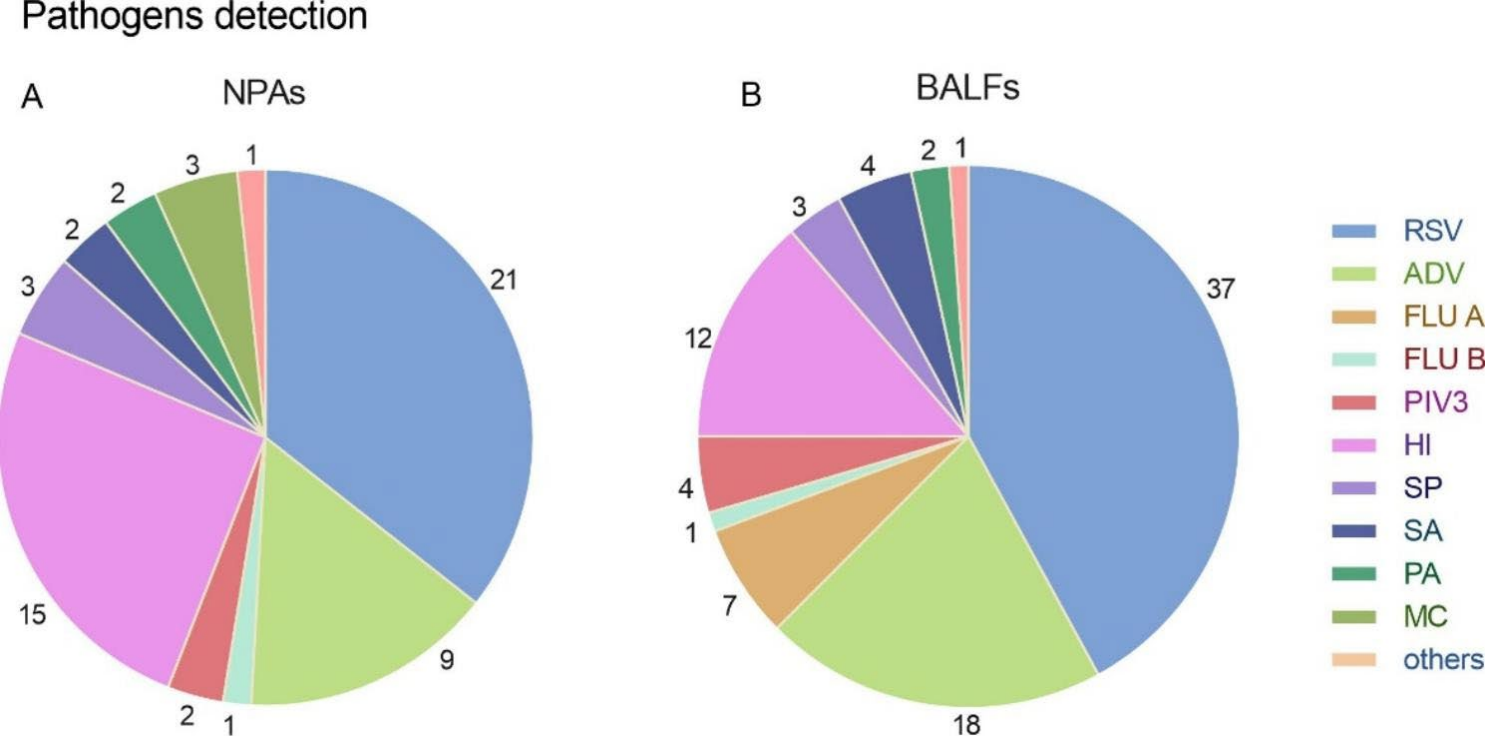
RSV and ADV were the two most frequently detetced viruses in both NPAs and BALFs. HI was the bacterium with the highest positive rate in both kind of samples

The positive rate of seven respiratory viruses in NPAs was 15.8% (33/204). RSV and ADV were the two dominate viruses, with positive rates of 10.2% (21/204) and 4.4% (9/204), respectively. A total of 12.7% (26/204) samples were found with bacteria positive, including 15 strains of HI, 3 strains of Streptococcus pneumoniae (SP), 3 strains of Moraxella catarrhalis (MC), 2 strains of Staphylococcus aureus (SA), 2 strains of Pseudomonas aeruginosa (PA) and one strain of Group A streptococcus (GAS). Mixture infection with viruses and bacteria were detected in 8 samples. The positive rate of pathogens in NPAs was 25.0% (51/204) (Fig. 2).

### Pathogens detected from BALFs

The positive rate of pathogens in BALFs was 36.7% (75/204) (Fig. 2). Respiratory viruses were found in 32.3% (66/204) of the BALFs. The positive rates of RSV, ADV, IV and PIV3 were 18.1% (37/204), 8.8% (18/204), 3.4% (7/204) and 1.9% (4/204), respectively. Bacteria pathogens were isolated from 10.7% (22/204) of the BALFs samples, including 12 strains of HI, 4 strains of SA, 3 strains of SP, 2 strains of PA and 1 strain of Escherichia coli (E. coli). There were 6.3% (13/204) of the samples with both bacteria and viruses detected.

### Comparison of pahtogens detection in NPAs and BALFs

**Fig. 3:**
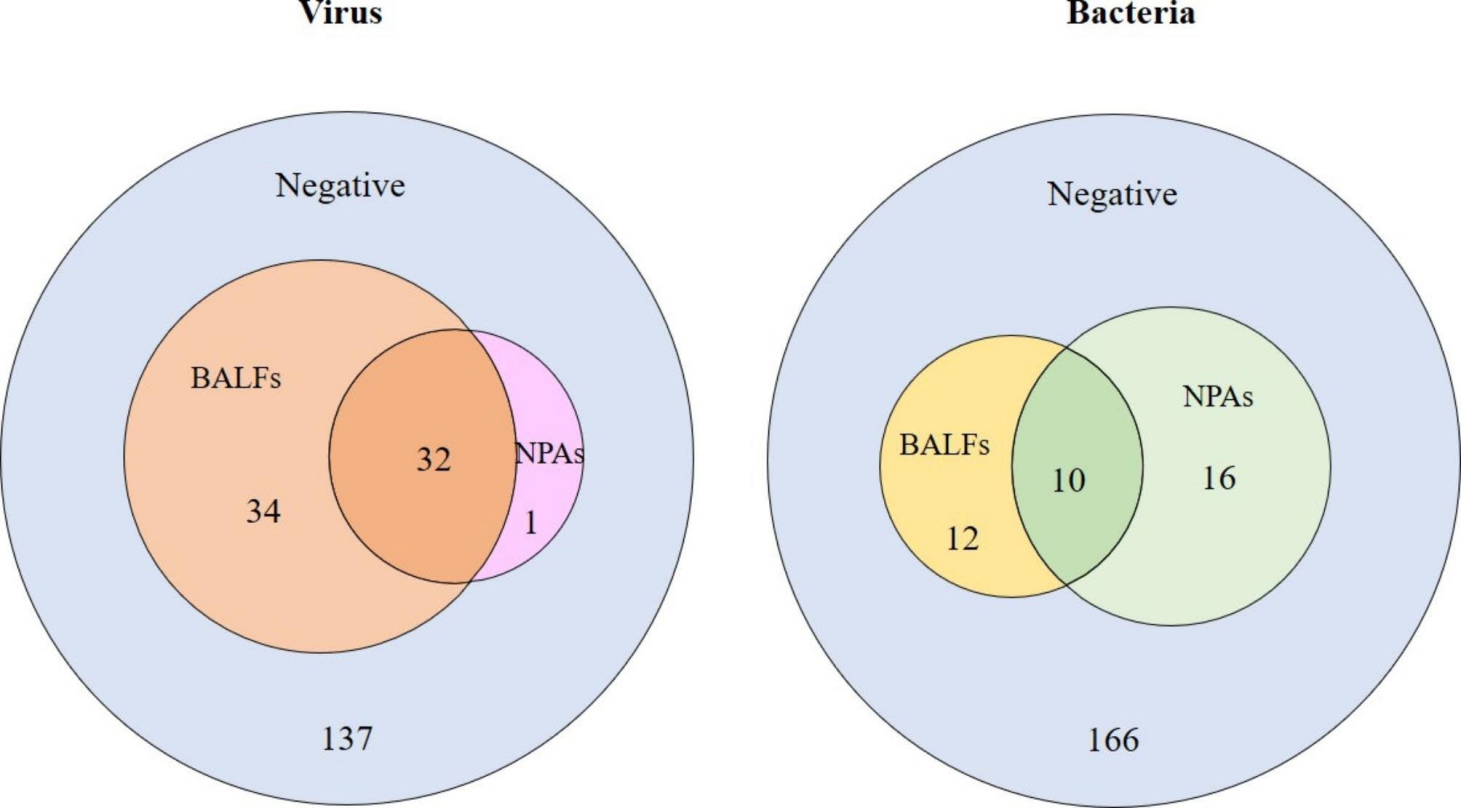
Higher rate of viruses were detected in BALFs compared to NPAs. However, minor difference of bacteria positive rates was observed between the two kind of samples

Higher rate of pathogens were detected in BALFs compared to NPAs, with positive rates of 36.7% (75/204) and 25.0% (51/204), respectively (x^2^ = 6.614, P = 0.010). Ninty-six piont 7% (32/33) of the viruses which detected in NPAs were also found in BALFs (Fig. 3). More respiratory viruses were detected in BALFs collected from those patients 3 days later. Taking the viruses detection in BALFs as the " golden standard”, the positive predictive value (PPV) of viruses detection in NPAs was 96.9% (32/33), and the negative predictive value (NPV) was 80.1% (137/171). Overall, the consistency of viruses detection between the two kind of samples was medium (kappa = 0.549).

**Fig. 4:**
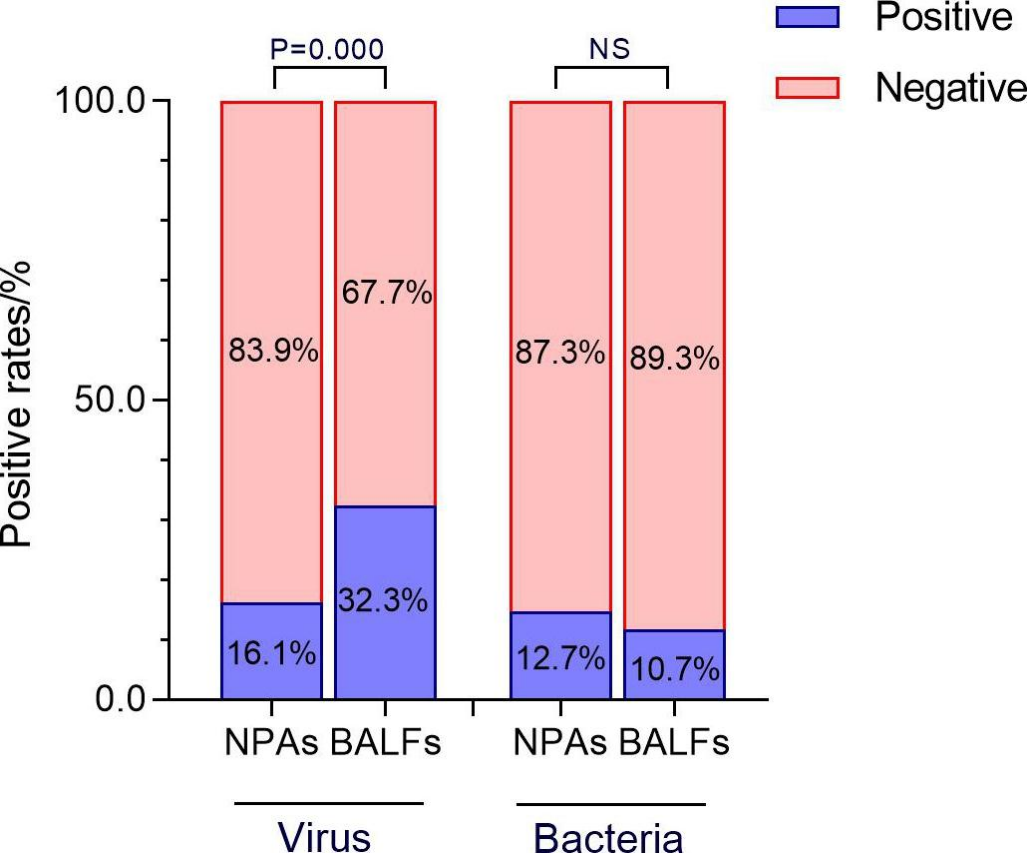
In NPAs and BALFs from the same patients, difference in the positive rates of respiratory virus was observed, but not in the positive rates of bacteria

The positive rate of bacterial in BALFs was 10.7% (22/204), lower than that of 12.7% (26/204) in NPAs. But there was no significant difference between the two kinds of samples (x^2^ = 0.378, *P* = 0.539) (Fig. 4). HI was the bacterium with the highest positive rate in both kind of samples, which was 7.3% (15/204) and 5.8% (12/ 204), respectively. Taking the bacteria detection in BALFs as the “golden standard”, the PPV of bacteria detection in NPAs was 38.4% (10/26), and the NPV was 93.2% (166/178). The consistency of bacteria detection between the two kind of samples was low, with kappa = 0.340. The detection of bacteria in two kind of samples was shown in Table 1.

**Table 1:** Detection of bacteria in NPAs and BALFs

NPAs /BALFs	HI	SP	SA	PA	MC	others	total
NPAs+	15	3	2	2	3	1	26
BALFs+	12	3	4	2	0	1	22
NPAs+/BALFs-	9	3	0	0	3	1	16
NPAs-/BALFs+	6	3	2	0	0	1	12
NPAs+/BALFs+	6	0	2	2	0	0	10
NPAs-/BALFs-	/	/	/	/	/	/	166
Kappa							0.340
PPV							38.4%
NPV							93.2%

HI was the bacterium with the highest positive rate in both kind of samples, but only 6 NPAs and 6 BALFs from the same case were both positive with HI.

## Discussion

By comparing the results of etiological detection from upper respiratory tract samples (NPAs) and BALFs, this study evaluated the reliability of upper respiratory tract samples used for diagnosis of lung pathogens in pediatric SCAPs, which would be attractiv to clinicians. In clinical setting, many pediatric cases developed to severe pneumonia even after receiving empiric anti-infective therapies [13–15].

Respiratory viruses are frequently detected in community-acquired pneumonia (CAP) among children of all ages globally [16–18]. The majority of pathogens associated with hospitalized SCAP in children were viruses in this study, which were consistent with the finding in this field over the past decade in high disease burden low and middle income countries (LMICs) as well as in Europe and the USA [11, 16, 19–23]. In this study, seven common respiratory viruses were found in 32.3% (66/204) of the BALFs tested by DFA, and such results were consistent with what we had seen over the past decade [12]. RSV and ADV were the two most frequently detected viruses from NPAs and BALFs.

Compared to BLAFs, only about half rate of respiratory viruses were detected in NPAs, and almost all the viruses detected in NPAs were also detected in BALFs. Using virus detection in BALFs as the “golden standard”, the positive predictive value (PPV) of viruses detection in NPAs was up to 96.9% (32/33), and the negative predictive value (NPV) was 80.1% (137/171). The results suggested that half of the seven viruses detected in NPAs by DFA could be missed, but the viruses that be found might be those presented in the lungs of SCAP cases.

HI was the most frequently isolated bacterium both in NPAs and BALFs, with positive rates of 7.3% (15/204) and 5.8% (12/ 204), respectively. Other common detected bacteria including SP, SA, PA and MC were also found [3, 6, 9, 13]. Such results were consistent with similar reports from Xiamen and other parts of the country [24, 25]. Both HI and SP were frequently detected, which indicates the importance of the promotion of vaccines in local younger children [26, 27]. We do not have the vaccine coverage data of HI type b (Hib) and SP because they are not included in China’s national immunization program. In a recent published paper, the researchers estimated annual national, regional, and provincial childhood mortality and morbidity attributable to Hib in 2010–2017 [28]. Although there was not significant difference in the positive rate of bacterial culture between NPAs and BALFs, we found that the inconsistency between the two kind of samples was obviously. Possible reasons for that discrepancy include: 1)Prior to the collection of BALFs, use of antibiotics possibly led to negative HI detection in BALFs. 2)HI had different meanings in the upper and lower respiratory tracts in those patients, or caused inflammation in different parts of the airway in the same case [29, 30]. As the length of the hospital stay increased, patient community-acquired Gram positive flora are increasingly replaced by hospital-acquired Gram negative flora, and the risk of hospital acquired pneumonia increases. This is why we chose to compare NPAs and BALFs collected within 3 days of each other. And we should mentioned that the lower bacterial potential pathogen detection in BALFs relative to NPAs might be partially contributed by nasopharyngeal colonisation with a wider range of flora than would be associated with invasive disease. Most invasive disease would be associated with SP, HI and SA, defining a lesser range of bacteria than colonised in the upper airway. Further, the types of infiltrative disease in the lower airway might also have influenced the type of bacterial pathogens detected. In a multi-site study using percutaneous lung aspirate (not BAL), SP predominated with lobar consolidation, whilst SA predominated in pleural effusion [10]. Not knowing the types of infiltrates appreciated on imaging scans, it is difficult to know how this might has affected the results of the present study.

Respiratory viruses and bacteria were co-detected in 3.9% (8/204) of the NPAs and 6.3% (13/204) of the BALFs, respectively. Based on our previous studies, the presence of co-pathogens or co-infections might have been significantly under-estimated, but that was not the point of this study [5, 18]. Our study proved that the detention results of NPAs were reliable in helping to identify the causes of lower respiratory tract infections when DFA was used to detect respiratory viruses in those cases. When routine bacterial culture was used for isolating the possible pathogens, inconsistent results might be presented while using NPAs and BALFs.

Our study has some limitations. Frist, in this single-center observational study, we selected SCAP pediatric patients who experienced bronchoscopy operations. The results of this study could not representthe cases with mild CAP or nosocomial pneumonia. Second, we did not use more sensitive molecular biological diagnostic methods, which might lead to low detention rates of pathogens [18, 31, 32]. Third, the human rhinovirus (HRV) was not studied which has become identified as a more prominent lower airway pathogen than previously suspected [5, 7, 9, 16, 17, 19, 21, 28, 30]. We still believed that our study had unique significances, given that the DFA method was based on the existent of viruses antigens judged by naked eyes with fluorescence microscope, and the routine bacterial culture, which are the two classical methods for pathogens detection. In addition, NPAs and BALFs in this study were not collected at the same time, which might lead to bias.

## Conclusion

The positive reports by DFA of the seven respiratory viruses from NPAs which collected within 3 days before bronchoscopy, could reflect the pathogens caused children SCAP. Results from NPAs and BALFs by routine bacterial culture might be inconsistent. and the selection of anti-infection strategies should be careful.

## Electronic supplementary material

Below is the link to the electronic supplementary material.

Supplementary Material 1: Original clinical data of 204 cases.

## Data Availability

The original contributions presented in the study are included in the article/Supplementary Material, further inquiries can be directed to the corresponding authors.

## Abbreviations

NPAs: nasopharyngeal aspirates.
BALFs: bronchoalveolar lavage fluids.
SCAP: severe community-acquired pneumonia.
PPV: positive predictive value.
NPV: negative predictive value.
DFA: direct immunofluorescence assay.
RSV: respiratory syncytial virus.
ADV: adenovirus.
IV: influenza virus.
PIV1-3: parainfluenza virus 1–3.
HI: Haemophilus influenzae.
SP: Streptococcus pneumoniae.
MC: Moraxella catarrhalis.
SA: Staphylococcus aureus.
PA: Pseudomonas aeruginosa.
GAS: Group A streptococcus.
E. coli: Escherichia coli.
